# Supplementary material for: lncRNA transcription induces meiotic recombination through chromatin remodelling in fission yeast
Source: Commun Biol. 2021 Mar 5;4:295. doi: 10.1038/s42003-021-01798-8 (PMC7935937; doi:10.1038/s42003-021-01798-8)
Supplement: Supplementary file 1 — Supplementary Information [file 42003_2021_1798_MOESM1_ESM.pdf]

Supplementary information

lncRNA transcription induces meiotic recombination through chromatin remodelling in fission yeast

Satoshi Senmatsu, Ryuta Asada, Arisa Oda, Charles S. Hoffman, Kunihiro Ohta, and Kouji Hirota

Supplementary Figure legends

Supplementary Fig. 1

Role of *mlon-IE*-induced transcription in meiotic recombination.

a, TSS of *ade6* transcripts induced from *mlon-IE* located in *M26:mlon-IE* cells during meiosis was determined by 5'-RACE. Black arrows indicate the TSS of *mlon-IE*-induced transcripts. The indicated number represents the position of the dominant TSS detected. The gray arrow indicates the TSS of the *ade6* transcript induced from the *M26* mutation. **b,** *ade6* transcript in *M26*, *M26::mlon-IE*, and *M26::mlon-IE-mut* cells during osmotic stress. Haploid cells were grown to a density of 2.0×10^7 cells/mL in YEL medium and transferred to YEL medium containing 1.2 M sorbitol. The cells were harvested 60 min after osmotic stress. **c,** *ade6* transcript in *M375* and *M375::mlon-IE* cells during osmotic stress.

Supplementary Fig. 2

mlon-IE*-induced transcription enhances meiotic recombination in *ade6-M26

a, Schematic representation of meiotic recombination in *ade6* gene. For the reconstitution of a functional *ade6*$^+$ gene, *M26::mlon-IE* cells require a longer recombination tract than do *M26* cells. Moreover, insertion of *mlon-IE* makes the distances between *M26::mlon-IE*/*469* shorter than that between *M26*/*469*. **b,** Insertion of *mlon-IE* leads to the expression of a nonfunctional Ade6 protein. Adenine prototropy was assessed by cell proliferation on SD plate medium lacking adenine.

Supplementary Fig. 3

mlon-IE*-induced transcription induces DSB and chromatin remodeling in *ade6-M26

a, Distribution of meiotic DSBs in *M26*, *M26::mlon-IE*, and *M26::mlon-IE-mut* cells. DSB band intensities around *M26* and *mlon-IE* 4 h after the onset of meiosis (dotted

line in Fig. 2b) were quantified using ImageJ (Plot Profile command). **b,** DSB intensities (dotted line in Fig. 2b) were quantified using ImageJ. DSB intensities normalized for parental band intensities relative to those of *M26* cells are presented. Error bars represent the standard deviation. n=3 biologically independent experiments. from three independent experiments. *P* values were calculated using unpaired one-sided Student's *t*-test: (***P* < .01). **c, d,** Histogram shows the quantification of MNase-sensitive bands at *M26* (c) (black arrowhead in Fig. 2c.) and *mlon-IE* (d) (dotted line in Fig. 2c). Error bars show the standard deviation. n=3 biologically independent experiments.

Supplementary Fig. 4

Genome-wide DSBs are not affected by the insertion of *mlon-IE* or *mlon-IE-mut* sequence

The genome-wide DSBs in the samples used in Fig. 2 and Fig. 3 were analyzed by pulsed-field gel electrophoresis as described in Methods.

Supplementary Fig. 5

***mlon-IE* plays a role downstream of transcription binding sequences created in *ade6-4002* and *ade6-4099* mutations**

a, Distribution of meiotic DSBs in *4002*, *4002::mlon-IE*, and *4002::mlon-IE-mut* cells. DSB band intensities around *4002* and *mlon-IE* 4 h after the onset of meiosis (dotted line in Fig. 3d) were quantified, as described in Supplementary Fig. 3a. **b,** DSB intensities (dotted line in Fig. 3d) normalized by parental band intensities relative to those of *4002* cells are presented. Error bars represent the standard deviation. n=3 biologically independent experiments. **c,** Meiotic DSB distribution in *4099*, *4099::mlon-IE*, and *4099::mlon-IE-mut* cells. DSB band intensities were quantified, as described in Supplementary Fig. 3a. **d,** DSB intensities (dotted line in Fig. 3e) were quantified, as described in Supplementary Fig. 3b. Error bars represent the standard

deviation. n=3 biologically independent experiments. *P* values were calculated using unpaired one-sided Student's *t*-test: (**$P < .01$).

Supplementary Fig. 6

Determination of a consensus sequence required for mlonRNA-c transcriptional initiation.

Northern blot analysis to examine *fbp1* transcripts in indicated cells carrying replacement sequences in *mlon-IE*. Cells were grown to a density of 2.0×10^7 cells/mL in YER medium and then transferred to YED medium. The cells were harvested at the indicated times. The *cam1* transcript was used as an internal control.

Supplementary Fig. 7

Chromatin remodeling mediated by *mlon-box*-dependent intergenic transcription in the *SPBC24C6.09c* upstream region

a, DSB intensities (dotted line in Fig. 5c) were quantified as described in Supplementary Fig. 3b. Distribution of meiotic DSBs in wild-type and *mlon-box-replacement* cells. DSB band intensity in the intergenic region between *SPBC24C6.09c* and *SPNCRNA.1506* at 4 h after the onset of meiosis (in Fig. 5c) was analyzed as described in Supplementary Fig. 3a. The arrow indicates DSB around *mlon-box*. **b,** Northern blot analysis in wild-type and *mlon-box-replacement* cells to examine *SPNCRNA.1506* transcription during *pat1-114*-induced meiosis. The right probe shown in Fig. 5b was used. 18S rRNA stained by ethidium bromide is shown as a loading control. **c**, Meiotic chromatin remodeling in indicated cells during *pat1-114*-induced meiosis. The black arrowhead and arrows indicate MNase-sensitive sites appearing meiotically around *mlon-box*.

Supplementary Fig. 8

mlon-box* in the *SPBC24C6.09c* upstream region regulates *SPBC24C6.09c

transcription in collaboration with Atf1

a, Schematic representation of *mlon-box* in the *SPBC24C6.09c* upstream region. The 10-nucleotide *mlon-box* sequence is shown in bold and italic, and the Atf1-binding-like motif is shaded. **b,** ChIP analysis to examine Atf1 binding at *mlon-box* in *SPBC24C6.09c* in indicated cells during *pat1-114*-induced meiosis. Error bars show the standard deviation. n=3 biologically independent experiments. *P* values were calculated using unpaired one-sided Student's *t*-test: (***P* < .01). **c,** Northern blot analysis to examine the *SPBC24C6.09c* transcript in indicated cells during *pat1-114*-induced meiosis. The *cam1* transcript is shown as a loading control.

Supplementary Fig. 9

***mlon-box* is enriched in the upstream region of the TSS**

Relationship between transcription start site (TSS) and *mlon-box*. The *X* axis indicates the distance from *mlon-box*, and the *Y* axis indicates the TSS frequency.

Supplementary Fig. 10

Uncropped images used in all figures in this study

Senmatsu et al.Supplementary Fig. 1

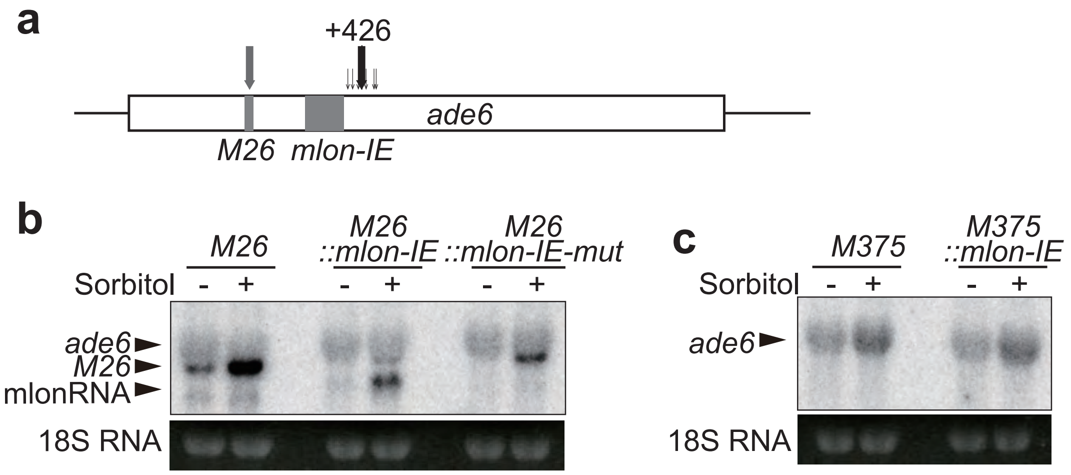

a

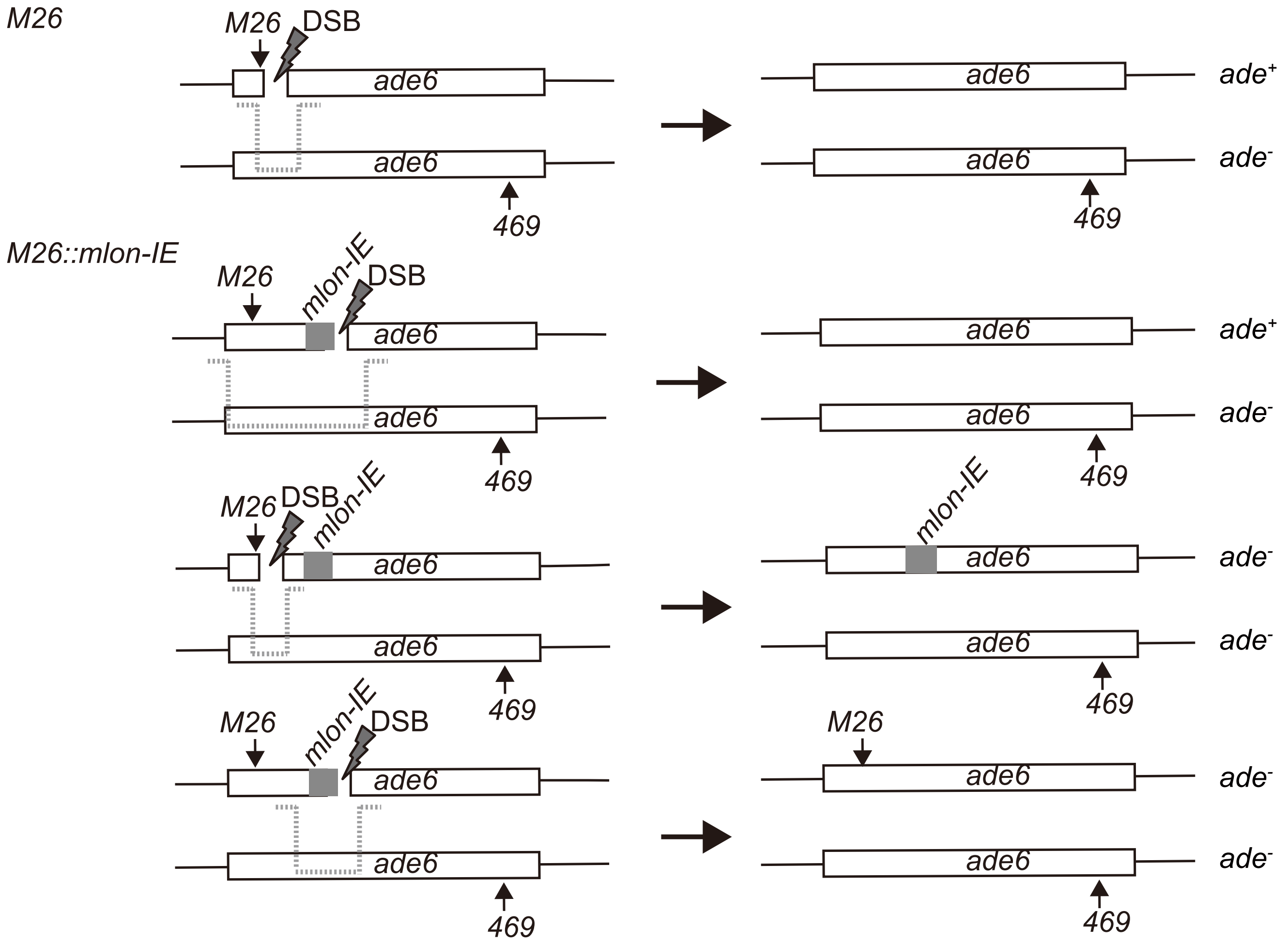
M26
M26
DSB
ade6
ade6
469
ade6
ade6
469
ade+
ade-
M26::mlon-IE
M26
mlon-IE
DSB
ade6
ade6
469
ade6
ade6
469
ade+
ade-
M26
DSB
mlon-IE
ade6
ade6
469
mlon-IE
ade6
ade6
469
ade-
ade-
M26
mlon-IE
DSB
ade6
ade6
469
M26
ade6
ade6
469
ade-
ade-

b

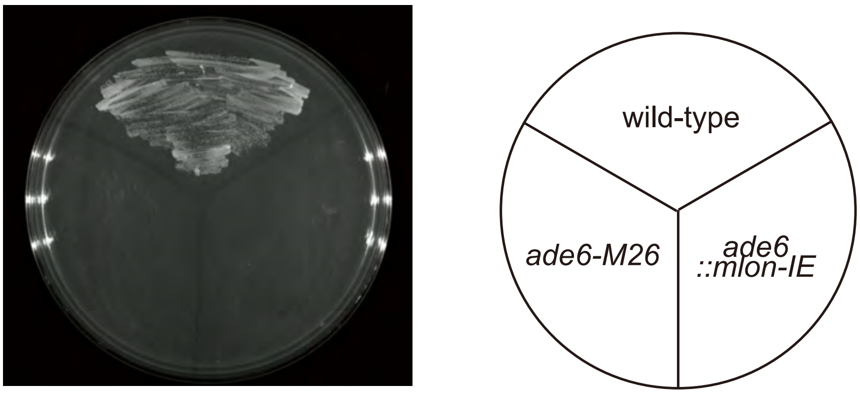
wild-type
ade6-M26
ade6
::mlon-IE

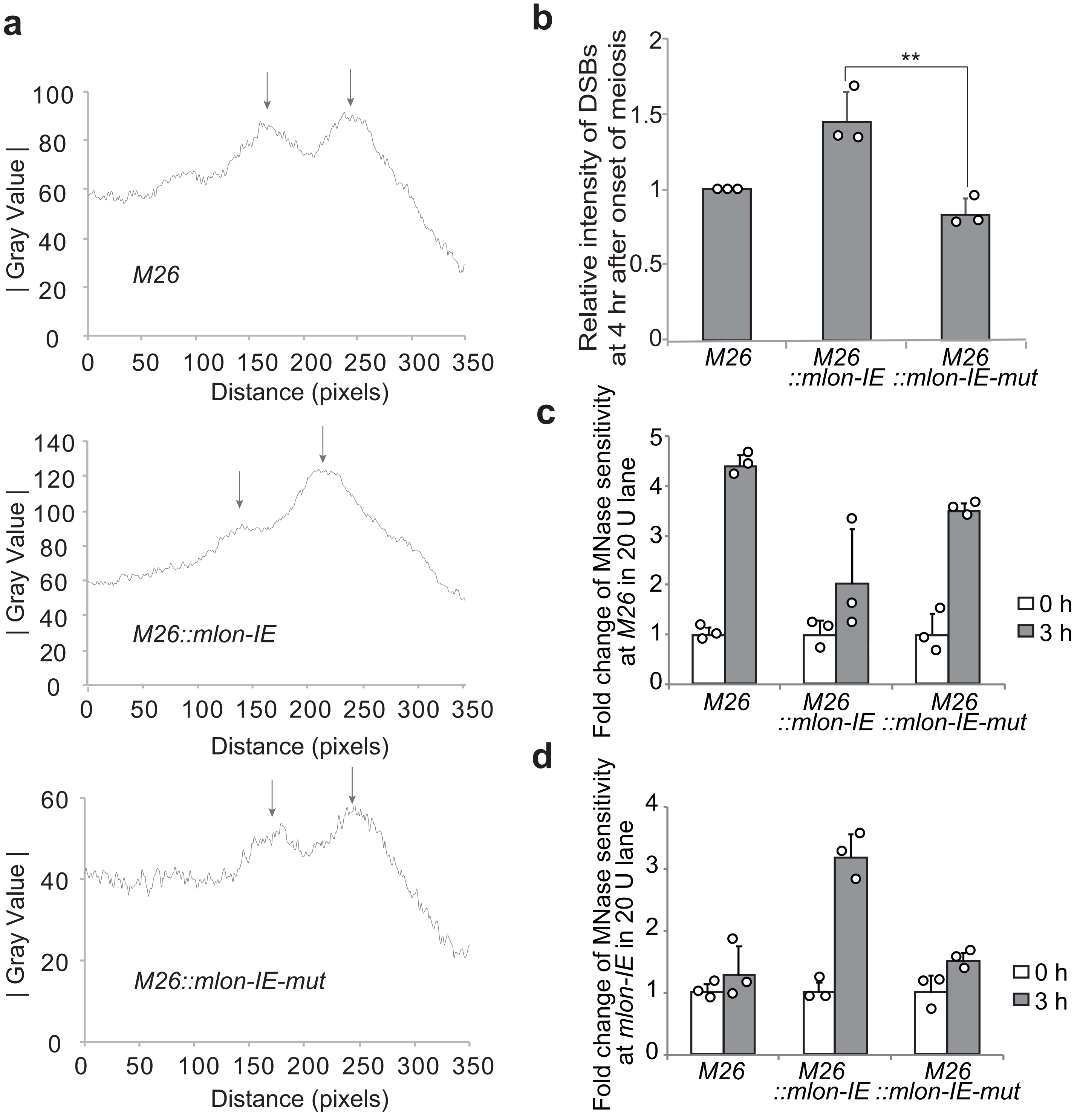
a
| Gray Value |
Distance (pixels)
M26
M26::mlon-IE
M26::mlon-IE-mut
b
Relative intensity of DSBs at 4 hr after onset of meiosis
**
M26
M26 ::mlon-IE
M26 ::mlon-IE-mut
c
Fold change of MNase sensitivity at M26 in 20 U lane
0 h
3 h
d
Fold change of MNase sensitivity at mlon-IE in 20 U lane

Senmatsu et al.Supplementary Fig. 4

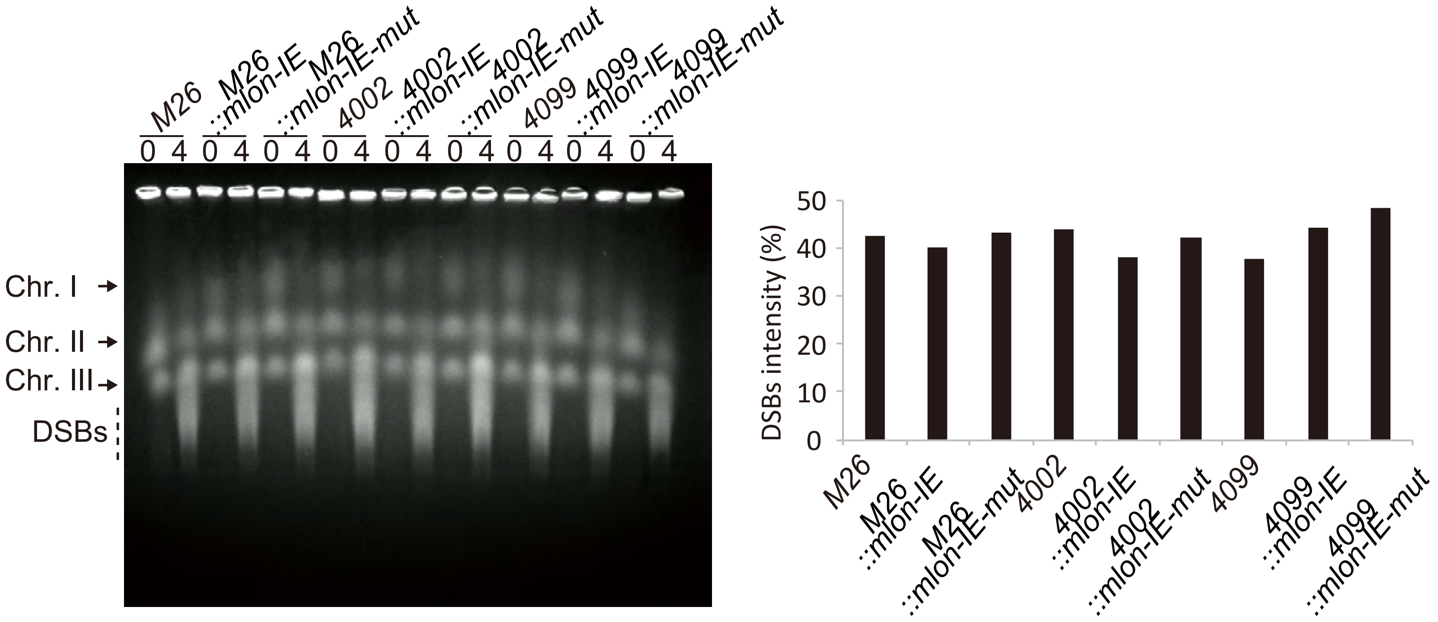

Senmatsu et al.Supplementary Fig. 5

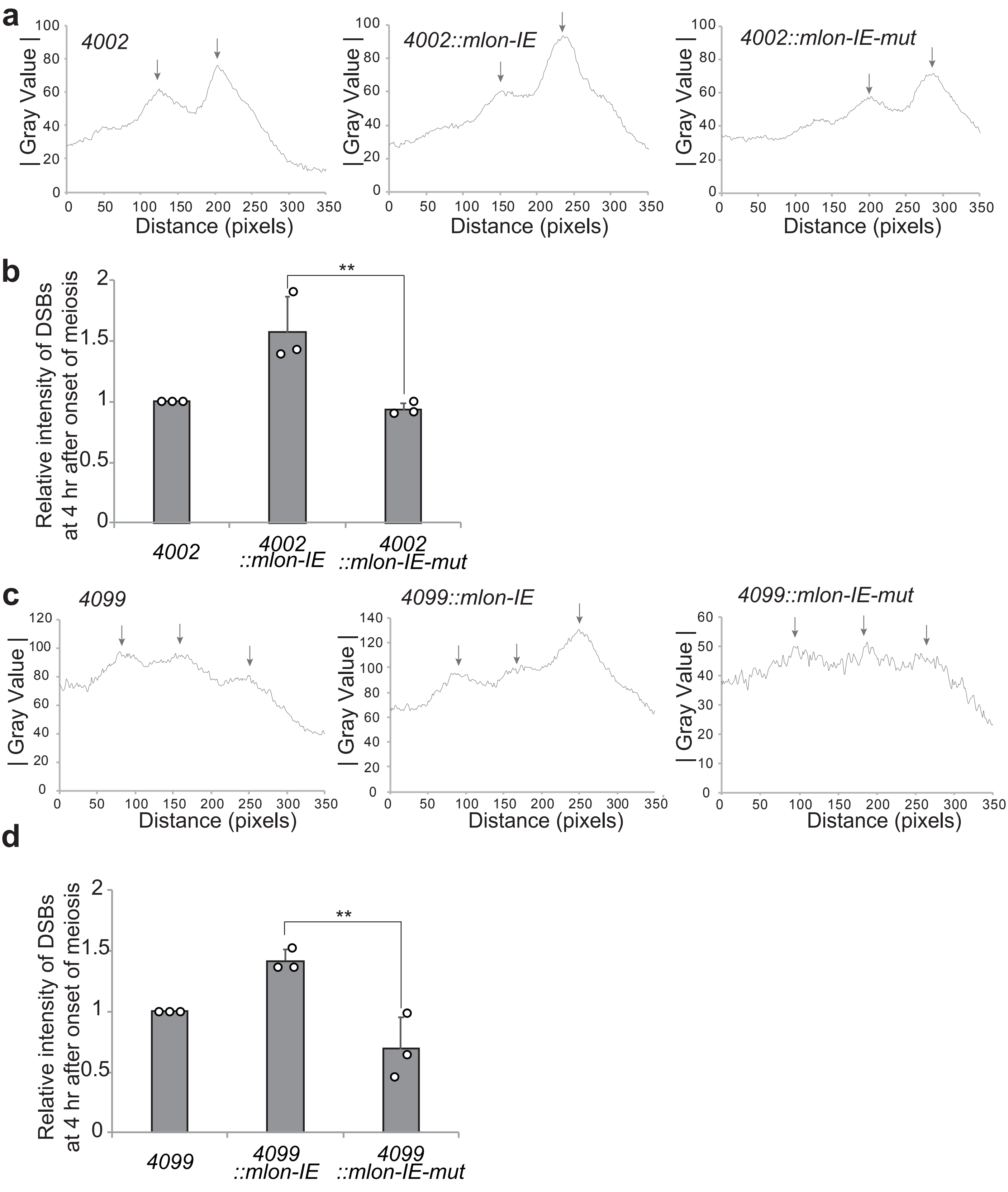

Senmatsu et al. Supplementary Fig. 6

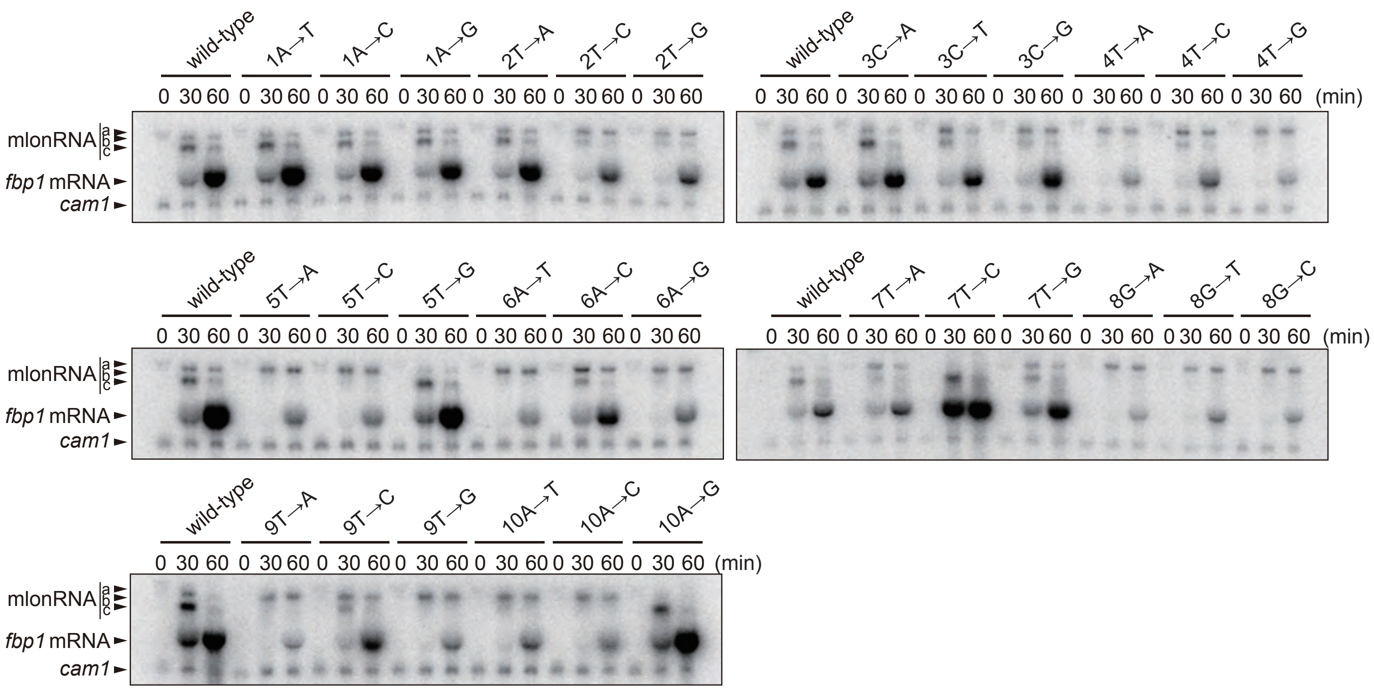

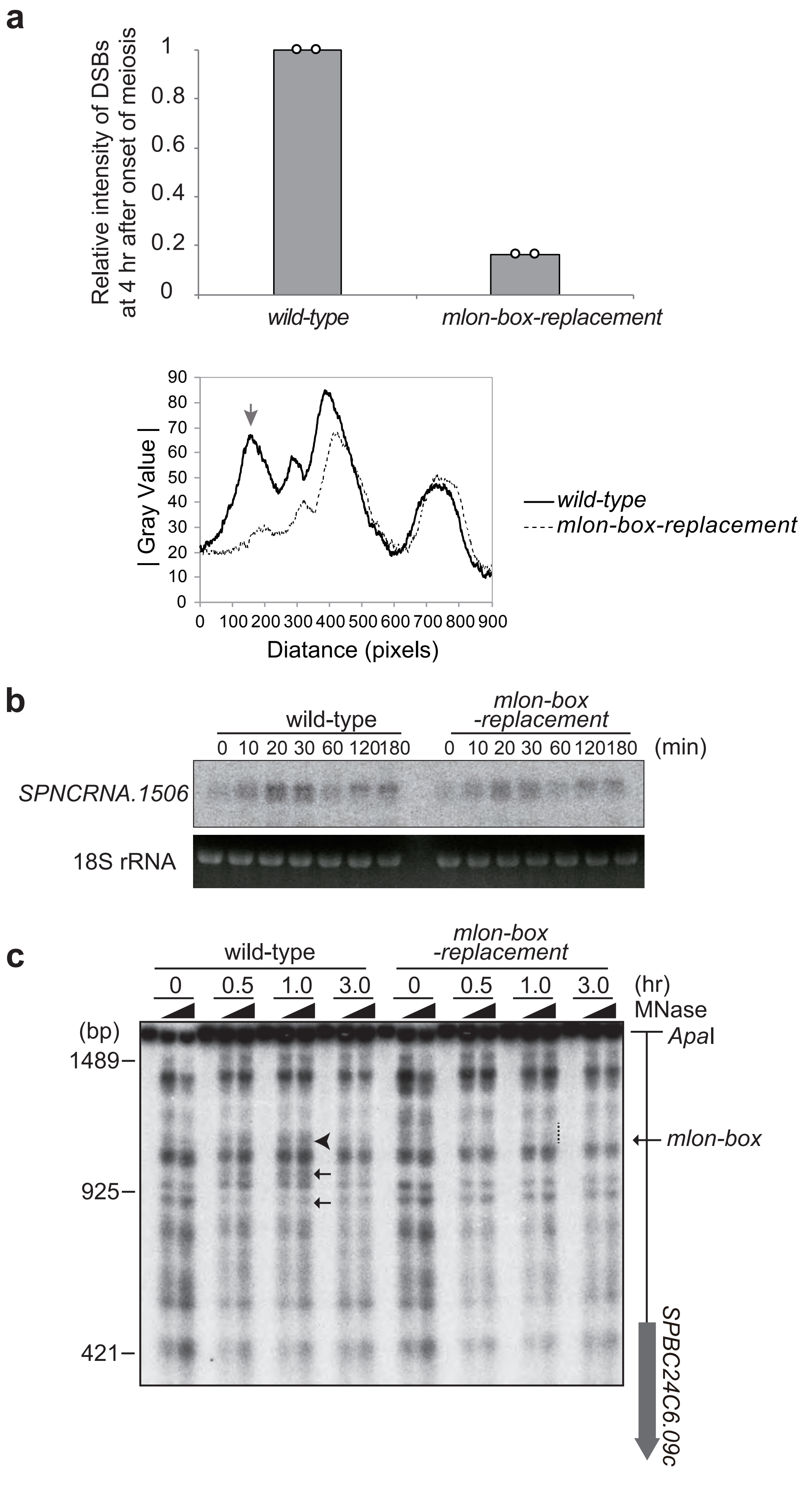
a
Relative intensity of DSBs at 4 hr after onset of meiosis
1
0.8
0.6
0.4
0.2
0
wild-type
mlon-box-replacement
Gray Value
90
80
70
60
50
40
30
20
10
0
0 100 200 300 400 500 600 700 800 900
Diatance (pixels)
wild-type
mlon-box-replacement
b
wild-type
mlon-box -replacement
0 10 20 30 60 120180 0 10 20 30 60 120180 (min)
SPNCRNA.1506
18S rRNA
c
wild-type
mlon-box -replacement
0 0.5 1.0 3.0 0 0.5 1.0 3.0 (hr)
MNase
(bp)
1489
925
421
ApaI
mlon-box
SPBC24C6.09c

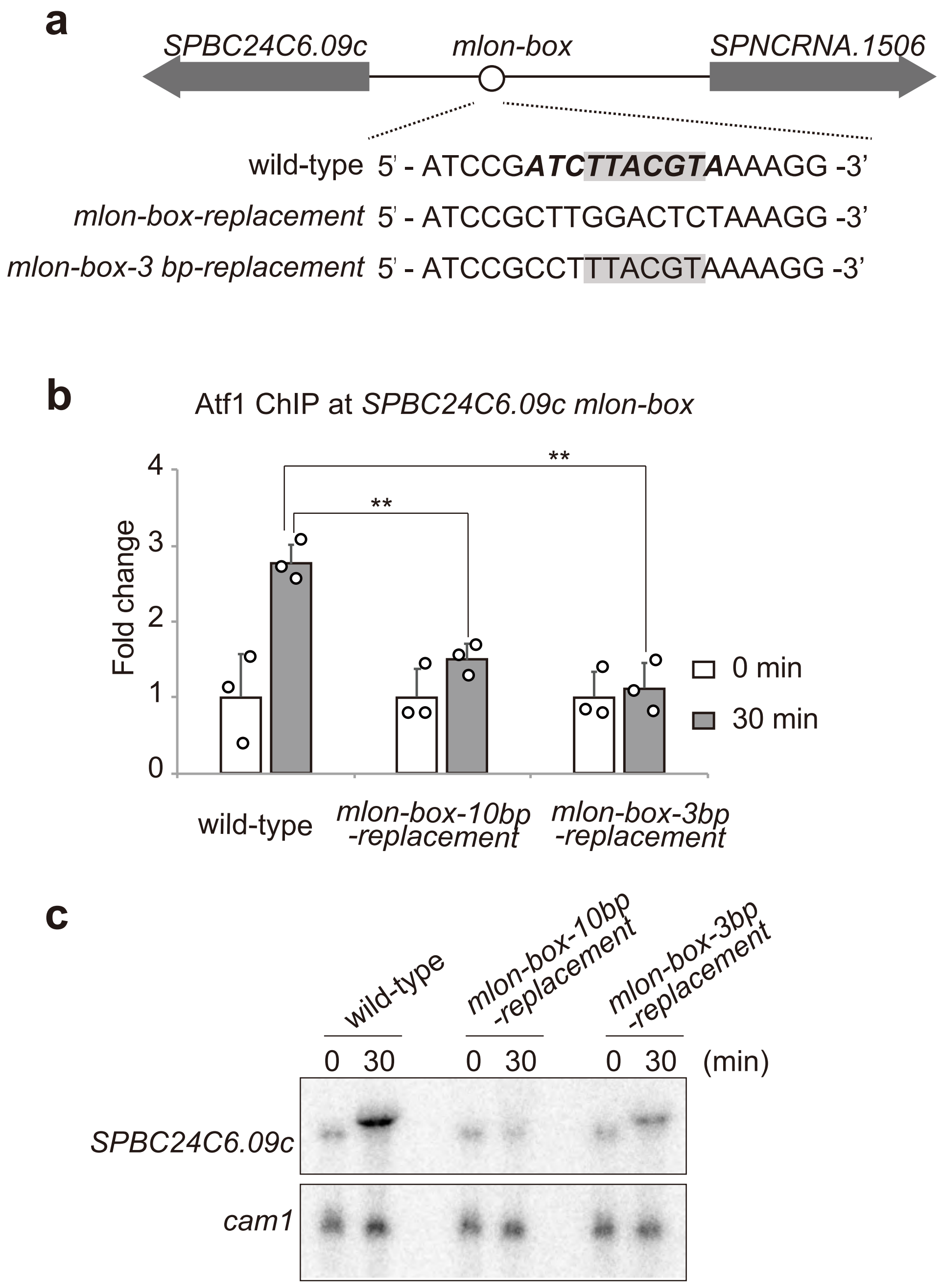
a
SPBC24C6.09c
mlon-box
SPNCRNA.1506
wild-type 5' - ATCCGATCTTACGTAAAAGG -3'
mlon-box-replacement 5' - ATCCGCTTGGACTCTAAAGG -3'
mlon-box-3 bp-replacement 5' - ATCCGCCTTTACGTAAAAGG -3'
b
Atf1 ChIP at SPBC24C6.09c mlon-box
Fold change
0
1
2
3
4
**
**
0 min
30 min
wild-type
mlon-box-10bp -replacement
mlon-box-3bp -replacement
c
wild-type
mlon-box-10bp -replacement
mlon-box-3bp -replacement
0 30 0 30 0 30 (min)
SPBC24C6.09c
cam1

Senmatsu et al. Supplementary Fig. 9

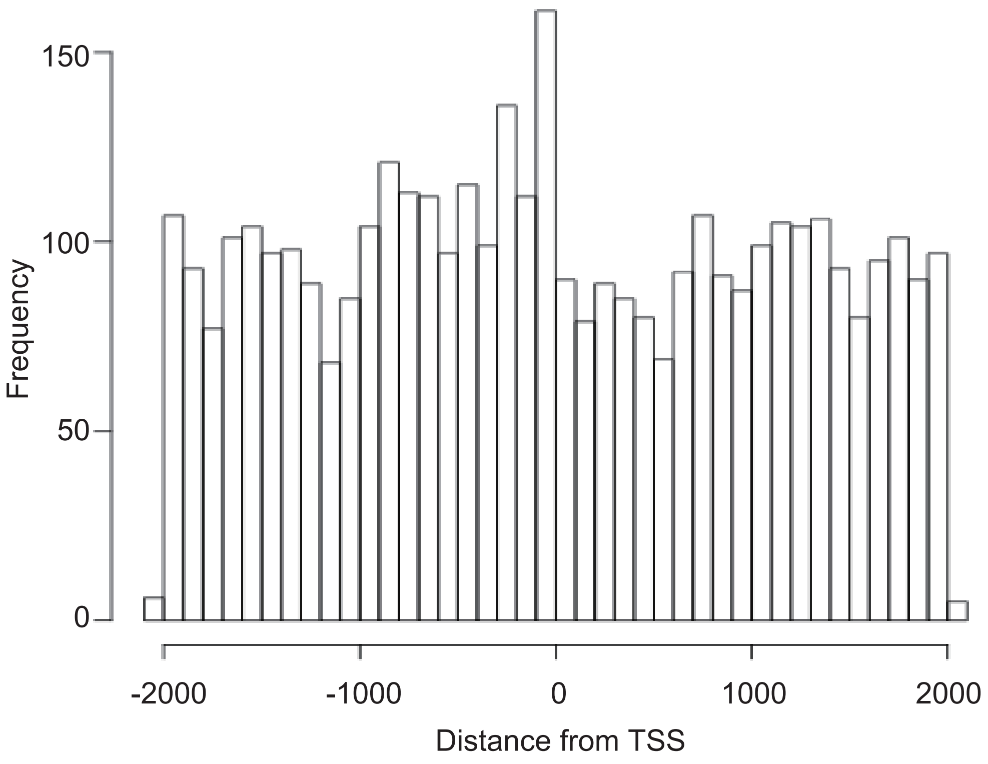

Senmatsu et al. Supplementary Fig. 10_1

Uncropped blot used in Fig. 1b

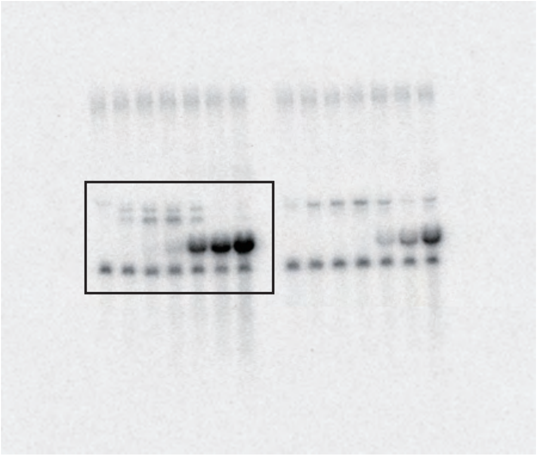

Uncropped blot used in Fig. 1b

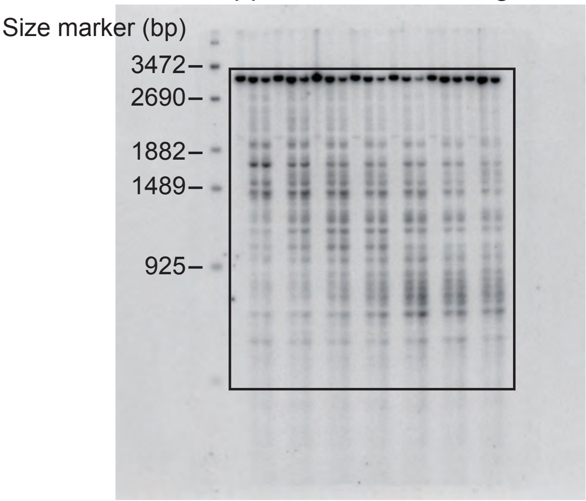

Uncropped blot used in Fig. 1d

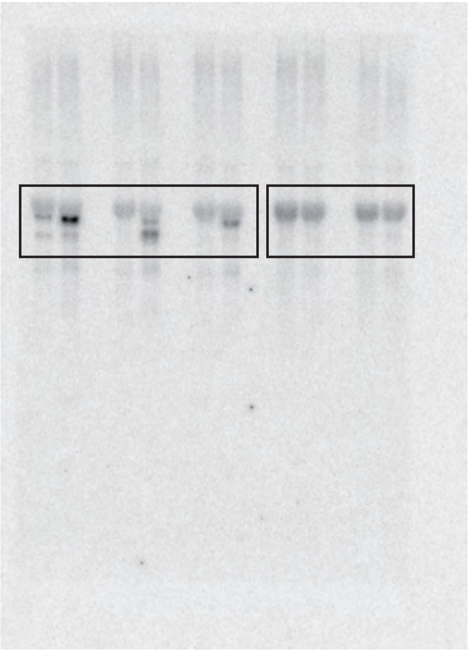

Uncropped gel used in Fig. 1d

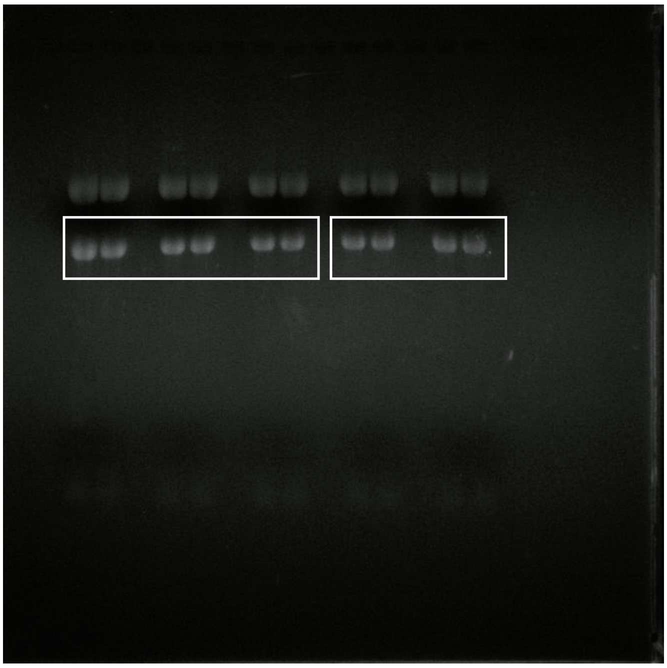

Senmatsu et al.Supplementary Fig. 10_2

Uncropped blot used in Fig. 2b

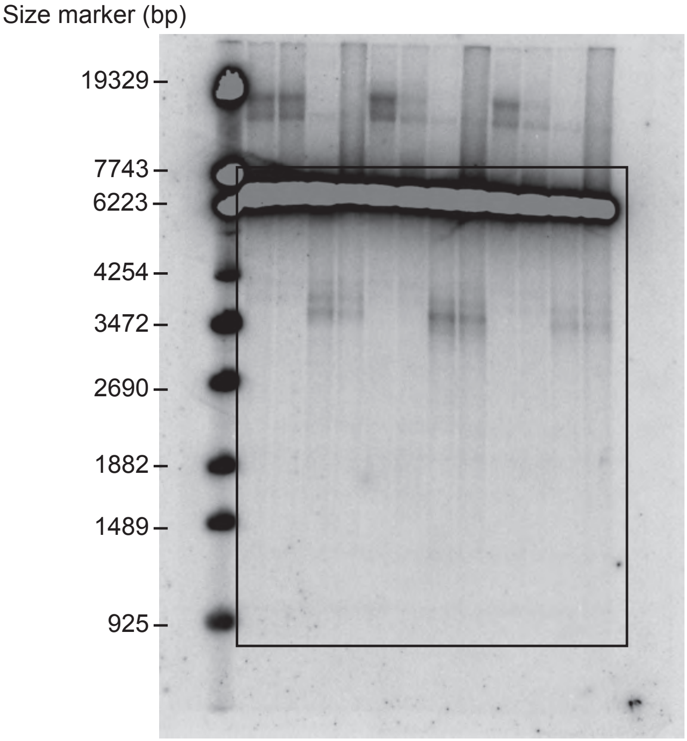

Uncropped blot used in Fig. 2c

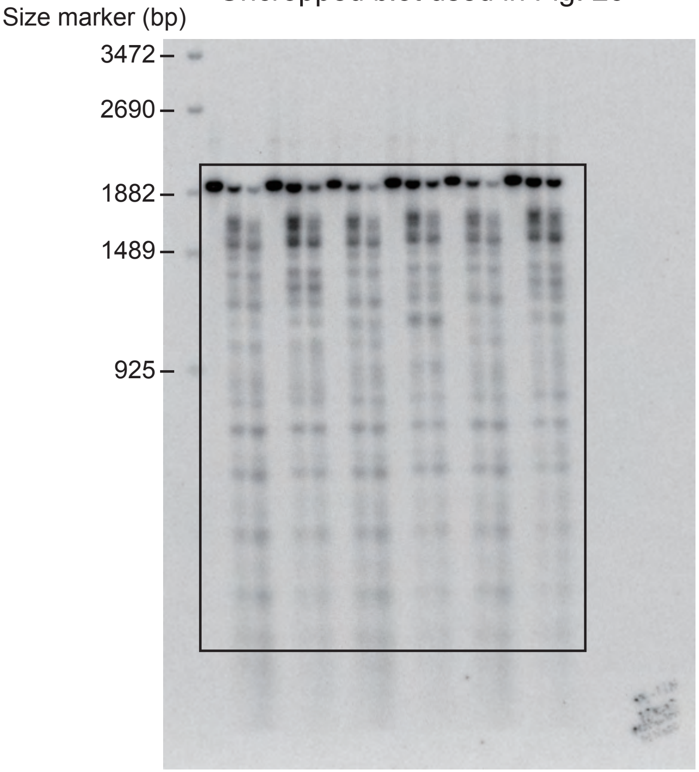

Senmatsu et al. Supplementary Fig. 10_3

Uncropped blot used in Fig. 3b

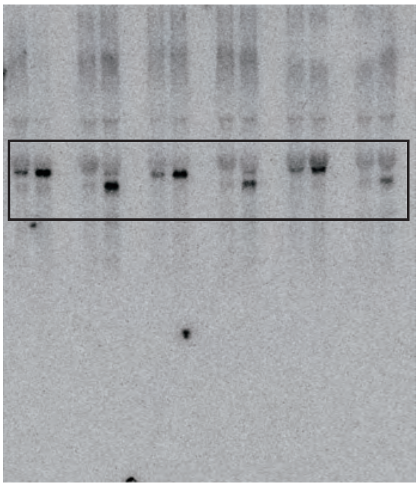

Uncropped gel used in Fig. 3b

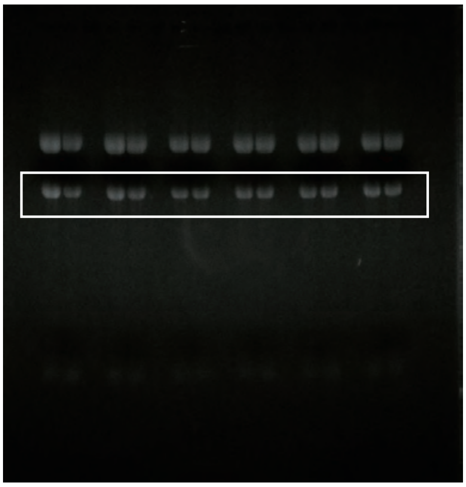

Uncropped blot used in Fig. 3b

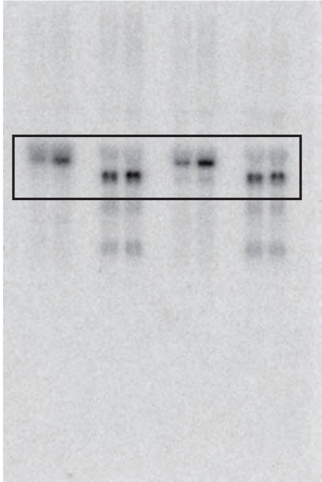

Uncropped gel used in Fig. 3b

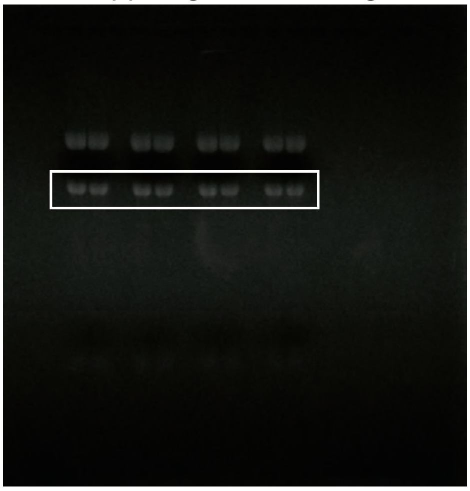

Uncropped blot used in Fig. 3d

Size marker (bp)

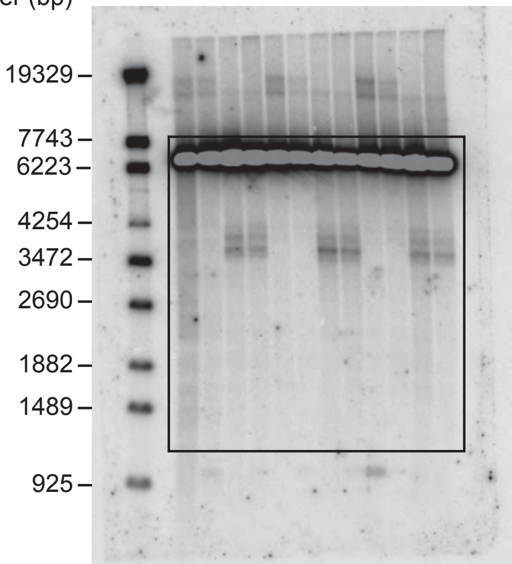

Uncropped blot used in Fig. 3e

Size marker (bp)

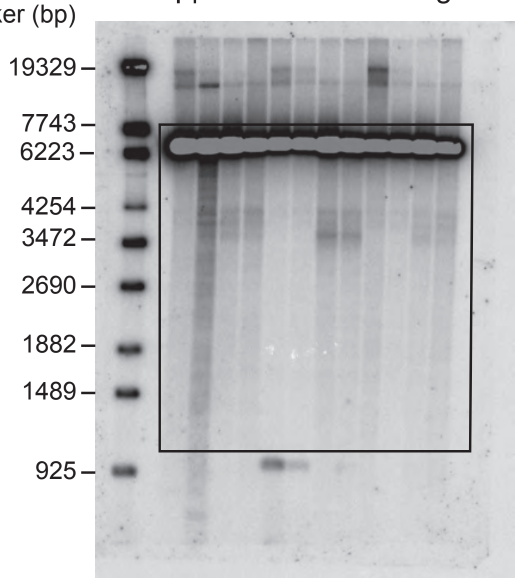

Senmatsu et al. Supplementary Fig. 10_4

Uncropped blot used in Fig. 5c

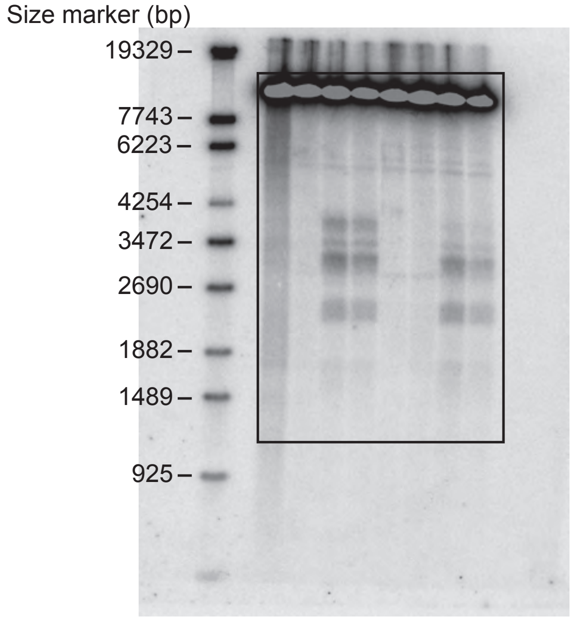

Uncropped blot used in Fig. 5d

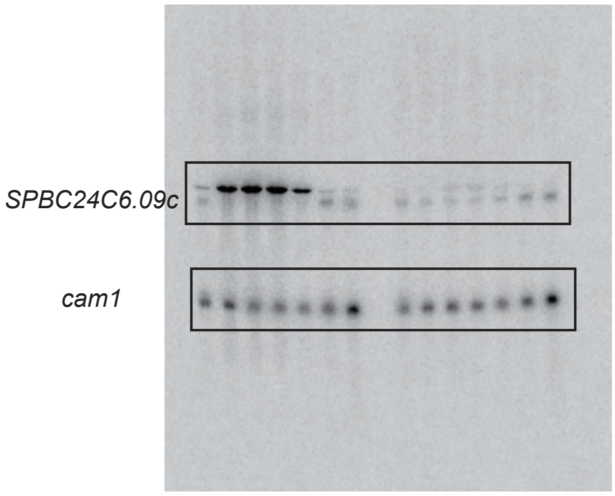

Uncropped blot used in Supplementary Fig. 1b, c

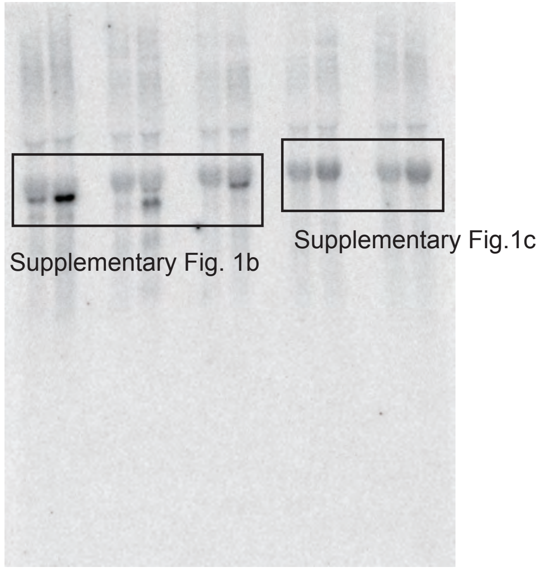

Uncropped gel used in Supplementary Fig. 1b, c

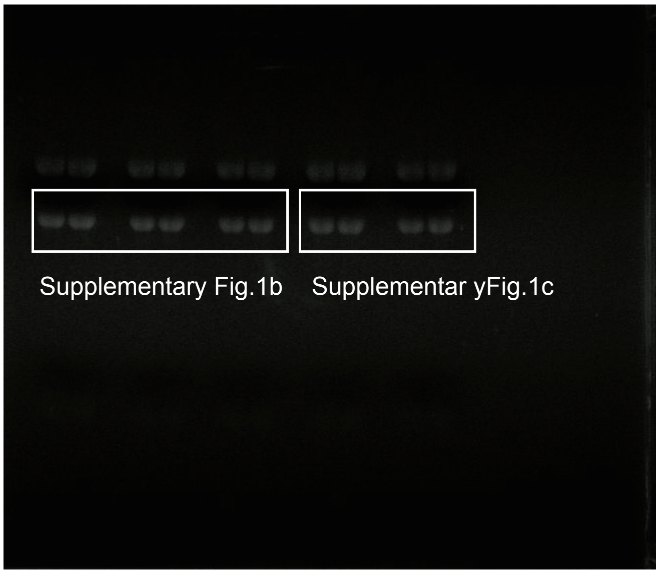

Senmatsu et al. Supplementary Fig. 10_5

Uncropped blot used in Supplementary Fig. 6

wild-type, replacement 1A and 2T

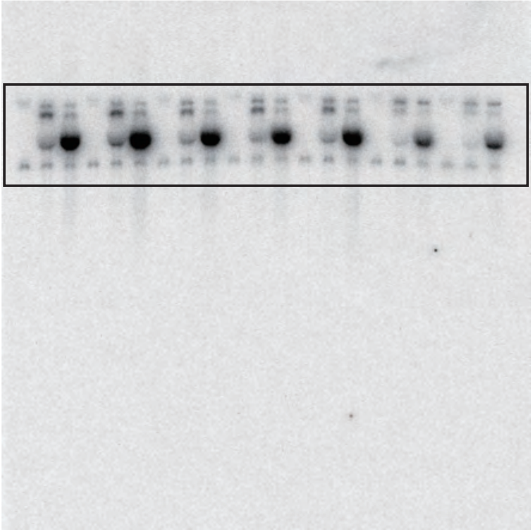

wild-type, replacement 3C and 4T

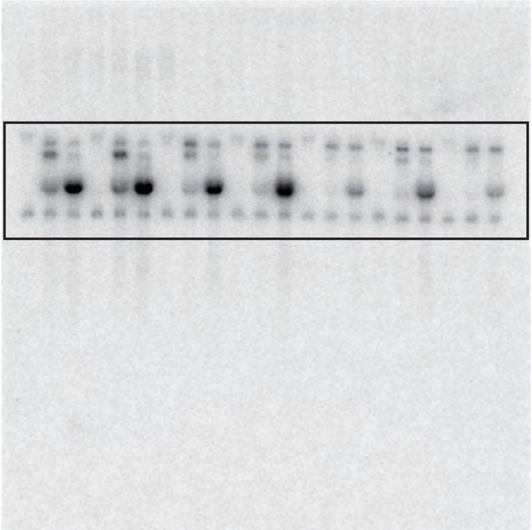

wild-type, replacement 5T and 6A

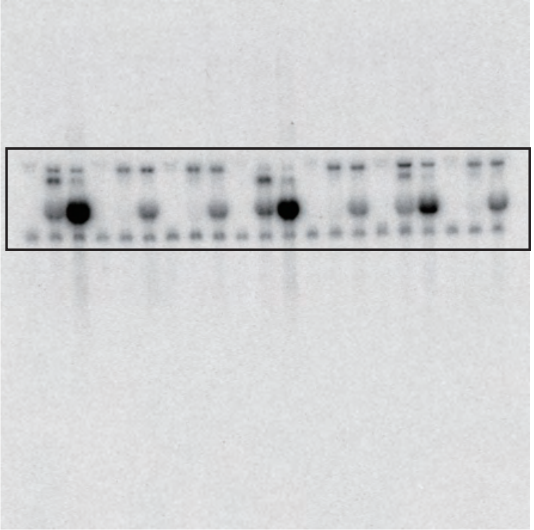

wild-type, replacement 7T and 8G

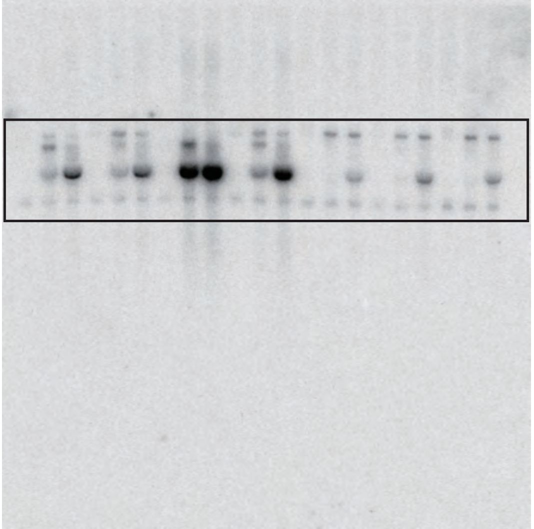

wild-type, replacement 9T and 10A

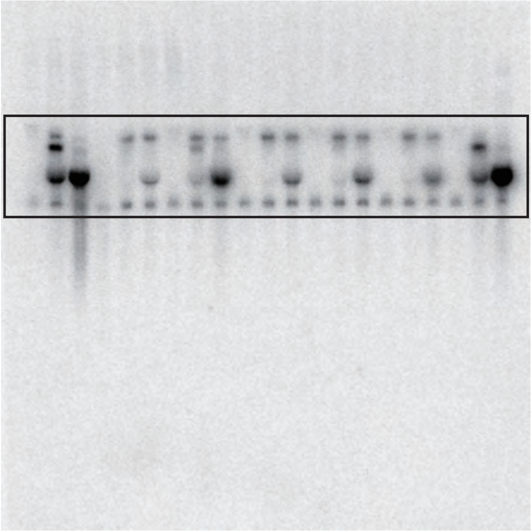

Uncropped blot used in Supplementary Fig. 7b

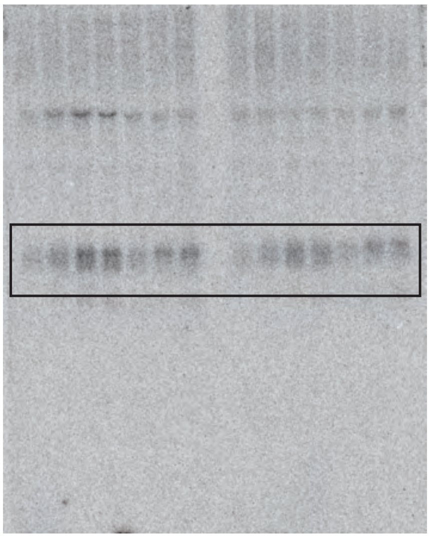

Uncropped gelt used in Supplementarya Fig. 7b

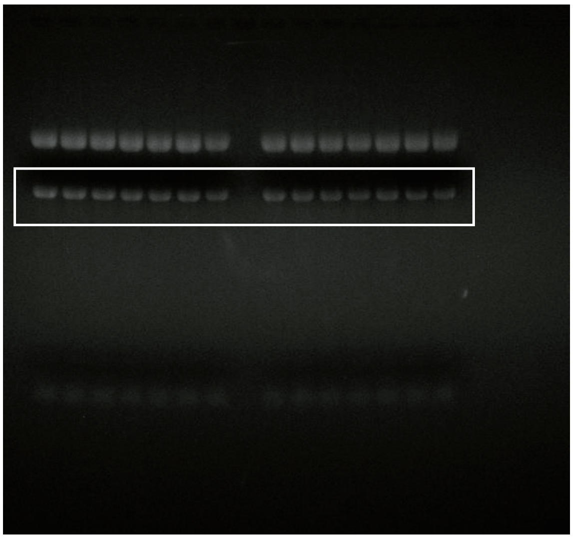

Uncropped blot used in Supplementary Fig. 7c

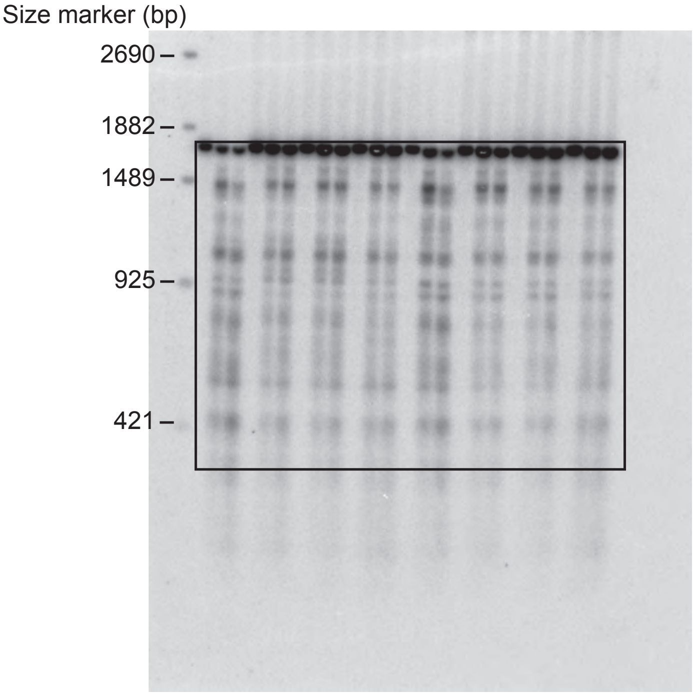

Uncropped blot used in Supplementary Fig. 8c

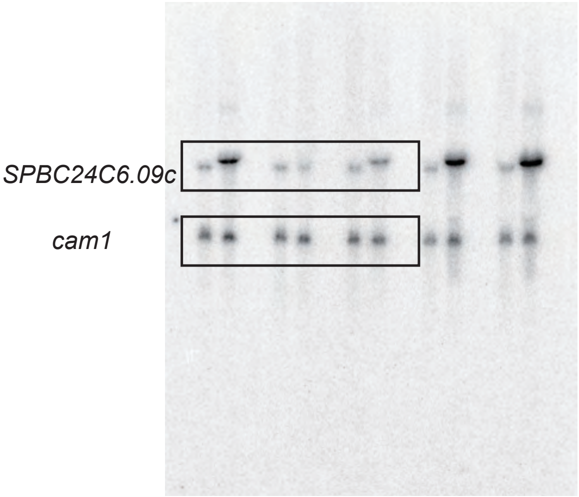

Supplementary Table 1

Strain	Genotype
SPH2	*h^+ ura4-D18*
SPH142	*h^+ ade6-M26 ura4-D18*
SPH143	*h^- ade6-469 leu1-32*
SPH313	*h^+ ura4-D18 mlonc promoter (ATCTTATGTA) :: (ATCTTATATA)*
SPH314	*h^+ ura4-D18 mlonc promoter (ATCTTATGTA) :: (ATCTTGTGTA)*
SPH521	*h^+ ura4-D18 mlonc promoter (ATCTTATGTA) :: (TTCTTATGTA)*
SPH522	*h^+ ura4-D18 mlonc promoter (ATCTTATGTA) :: (CTCTTATGTA)*
SPH523	*h^+ ura4-D18 mlonc promoter (ATCTTATGTA) :: (GTCTTATGTA)*
SPH524	*h^+ ura4-D18 mlonc promoter (ATCTTATGTA) :: (AACTTATGTA)*
SPH525	*h^+ ura4-D18 mlonc promoter (ATCTTATGTA) :: (ACCTTATGTA)*
SPH526	*h^+ ura4-D18 mlonc promoter (ATCTTATGTA) :: (AGCTTATGTA)*
SPH527	*h^+ ura4-D18 mlonc promoter (ATCTTATGTA) :: (ATATTATGTA)*
SPH528	*h^+ ura4-D18 mlonc promoter (ATCTTATGTA) :: (ATTTTATGTA)*
SPH529	*h^+ ura4-D18 mlonc promoter (ATCTTATGTA) :: (ATGTTATGTA)*
SPH530	*h^+ ura4-D18 mlonc promoter (ATCTTATGTA) :: (ATCATATGTA)*
SPH531	*h^+ ura4-D18 mlonc promoter (ATCTTATGTA) :: (ATCCTATGTA)*
SPH532	*h^+ ura4-D18 mlonc promoter (ATCTTATGTA) :: (ATCGTATGTA)*
SPH533	*h^+ ura4-D18 mlonc promoter (ATCTTATGTA) :: (ATCTAATGTA)*
SPH534	*h^+ ura4-D18 mlonc promoter (ATCTTATGTA) :: (ATCTCATGTA)*
SPH535	*h^+ ura4-D18 mlonc promoter (ATCTTATGTA) :: (ATCTGATGTA)*
SPH536	*h^+ ura4-D18 mlonc promoter (ATCTTATGTA) :: (ATCTTTTGTA)*

SPH537 *h⁺ ura4-D18 mlonc promoter (ATCTTATGTA) :: (ATCTTGTGTA)*

SPH538 *h⁺ ura4-D18 mlonc promoter (ATCTTATGTA) :: (ATCTTAAGTA)*

SPH539 *h⁺ ura4-D18 mlonc promoter (ATCTTATGTA) :: (ATCTTACGTA)*

SPH540 *h⁺ ura4-D18 mlonc promoter (ATCTTATGTA) :: (ATCTTAGGTA)*

SPH541 *h⁺ ura4-D18 mlonc promoter (ATCTTATGTA) :: (ATCTTATTTA)*

SPH542 *h⁺ ura4-D18 mlonc promoter (ATCTTATGTA) :: (ATCTTATCTA)*

SPH543 *h⁺ ura4-D18 mlonc promoter (ATCTTATGTA) :: (ATCTTATGAA)*

SPH544 *h⁺ ura4-D18 mlonc promoter (ATCTTATGTA) :: (ATCTTATGCA)*

SPH545 *h⁺ ura4-D18 mlonc promoter (ATCTTATGTA) :: (ATCTTATGGA)*

SPH546 *h⁺ ura4-D18 mlonc promoter (ATCTTATGTA) :: (ATCTTATGGT)*

SPH547 *h⁺ ura4-D18 mlonc promoter (ATCTTATGTA) :: (ATCTTATGGC)*

SPH548 *h⁺ ura4-D18 mlonc promoter (ATCTTATGTA) :: (ATCTTATGGG)*

SPH551 *h⁺ ura4-D18 ade6-M26(+313 to +377)::fbp1(-870 to 806)*

SPH556 *h⁺ ura4-D18 ade6-M26(+313 to +377)::fbp1(-870 to 806, (-851 to -842)::act1(+456 to +465))*

SPH574 *h⁺ ura4-D18 ade6-M26(+313 to +377)::fbp1(-870 to 806) his3-D1*

SPH583 *h⁺ ura4-D18 ade6-M26 his3-D1*

SPH584 *h⁺/h⁻ ade6-M26(+313 to +377)::fbp1(-870 to 806)/ade6-M26(+313 to +377)::fbp1(-870 to 806) leu1-32/+ his3-D1/+ ura4-D18/ura4-D18*

SPH585 *h⁺/h⁻ ade6-M26/ade6-M26 leu1-32/+ his3-D1/+ ura4-D18/ura4-D18*

SPH586 *h⁺ ura4-D18 ade6-M375*

SPH587 *h⁺ ura4-D18 ade6-M375(+313 to +377)::fbp1(-870 to 806)*

SPH595 *h⁺ ura4-D18 ade6-M26(+313 to +377)::fbp1(-870 to 806, (-851 to -842)::act1(+456 to +465)) his3-D1*

SPH597 h^+ *ura4-D18 ade6-M375 his3-D1*

SPH603 h^+/h^- *ade6-M26(+313 to +377)::fbp1(-870 to 806, (-851 to -842)::act1(+456 to +465))/ade6-M26(+313 to +377)::fbp1(-870 to 806, (-851 to -842)::act1(+456 to +465)) leu1-32/+ his3-D1/+ ura4-D18/ura4-D18*

SPH604 h^+/h^- *ade6-M375/ade6-M375 leu1-32/+ his3-D1/+ ura4-D18/ura4-D18*

SPH606 h^+ *ura4-D18 ade6-M375(+313 to +377)::fbp1(-870 to 806)*

SPH610 h^+ *ade6-M26 ura4-D18 pat1-114 rad50S*

SPH617 h^+ *ura4-D18 ade6-M375(+313 to +377)::fbp1(-870 to 806) his3-D1*

SPH621 h^+ *ura4-D18 ade6-4002(+313 to +377)::fbp1(-870 to 806) his3-D1*

SPH623 h^+ *ura4-D18 ade6-4099(+313 to +377)::fbp1(-870 to 806) his3-D1*

SPH628 h^+/h^- *ade6-M375(+313 to +377)::fbp1(-870 to 806)/ade6-M375(+313 to +377)::fbp1(-870 to 806) leu1-32/+ his3-D1/+ ura4-D18/ura4-D18*

SPH631 h^+/h^- *ade6-4002(+313 to +377)::fbp1(-870 to 806)/ade6-4002(+313 to +377)::fbp1(-870 to 806) leu1-32/+ his3-D1/+ ura4-D18/ura4-D18*

SPH632 h^+/h^- *ade6-4099(+313 to +377)::fbp1(-870 to 806)/ade6-4099(+313 to +377)::fbp1(-870 to 806) leu1-32/+ his3-D1/+ ura4-D18/ura4-D18*

SPH633 h^+/h^- *ade6-4095(+313 to +377)::fbp1(-870 to 806)/ade6-4095(+313 to +377)::fbp1(-870 to 806) leu1-32/+ his3-D1/+ ura4-D18/ura4-D18*

SPH634 h^+/h^- *ade6-4156(+313 to +377)::fbp1(-870 to 806)/ade6-4156(+313 to +377)::fbp1(-870 to 806) leu1-32/+ his3-D1/+ ura4-D18/ura4-D18*

SPH640 h^+/h^- *ade6-M26(+323 to +352)::fbp1(-860 to 831)/ ade6-M26(+323 to +352)::fbp1(-860 to 831) leu1-32/+ his3-D1/+ ura4-D18/ura4-D18*

SPH641 h^+/h^- *ade6-M26(+333 to +342)::fbp1(-850 to 841)/ ade6-M26(+333 to +342)::fbp1(-850 to 841) leu1-32/+ his3-D1/+ ura4-D18/ura4-D18*

SPH646 h^+ *ade6-M26 ura4-D18 SPBC24C6.09c promoter(-614 to -605)::act1(+465 to +456) pat1-114 rad50S*

SPH649 h^+ *ade6-M26(+313 to +377)::fbp1(-870 to 806) ura4-D18 pat1-114 rad50S*

SPH658 h^+/h^- *ade6-4002/ade6-4002 leu1-32/+ his3-D1/+ ura4-D18/ura4-D18*

SPH659 h^+/h^- *ade6-4099/ade6-4099 leu1-32/+ his3-D1/+ ura4-D18/ura4-D18*

SPH660 h^+/h^- *ade6-4095/ade6-4095 leu1-32/+ his3-D1/+ ura4-D18/ura4-D18*

SPH661 h^+/h^- *ade6-4156/ade6-4156 leu1-32/+ his3-D1/+ ura4-D18/ura4-D18*

SPH673 h^+ *ade6-M26(+313 to +377)::fbp1(-870 to 806, (-851 to -842)::act1(+456 to +465)) ura4-D18 pat1-114 rad50S*

SPH703 h^+ *ura4-D18 ade6-4002(+313 to +377)::fbp1(-870 to 806, (-851 to -842)::act1(+456 to +465)) his3-D1*

SPH706 h^+ *ura4-D18 ade6-M26(+313 to +377)::fbp1(-870 to 806, (-851 to -842)::act1(+456 to +465)) his3-D1*

SPH715 h^+ *ura4-D18 ade6-4099(+313 to +377)::fbp1(-870 to 806, (-851 to -842)::act1(+456 to +465)) his3-D1*

SPH742 h^+ *ade6-4002 ura4-D18 pat1-114 rad50S*

SPH743 h^+ *ade6-4002(+313 to +377)::fbp1(-870 to 806) ura4-D18 pat1-114 rad50S*

SPH744	*h^+ ade6-4002(+313 to +377)::fbp1(-870 to 806, (-851 to -842)::act1(+456 to +465)) ura4-D18 pat1-114 rad50S*
SPH745	*h^+ ade6-4099 ura4-D18 pat1-114 rad50S*
SPH746	*h^+ ade6-4099(+313 to +377)::fbp1(-870 to 806) ura4-D18 pat1-114 rad50S*
SPH747	*h^+ ade6-4099(+313 to +377)::fbp1(-870 to 806, (-851 to -842)::act1(+456 to +465)) ura4-D18 pat1-114 rad50S*
SPH811	*h^+ ade6-M26 ura4-D18 SPBC24C6.09c promoter(ATCTTACGTA)::(CCTTTACGTA) pat1-114 rad50S*

Supplementary Table 2

No.	Name	Sequence (5'-3')
p1	ade6-M26 cloning Fw	AAAAACTAGTTGTCGAGTTCCTCTTGAAAC
p2	ade6-mlonc-1	TTCATCACCGACCTCAATTGCATACCCACAGC AATTACATAAGATCGACCCCAATTGCCTATGA TTTTGTCTTGAATGCATCGCAGA
p3	ade6-M26 cloning Rev	AAAAGGGCCCGCAAGTATACGACAGGCTAA
p4	ade6-mlonc-2	ATCATAGGCAATTGGGGTCGATCTTATGTAAT TGCTGTGGGTATGCAATTGAGGTCGGTGATGA ACCAGGAAAGTGTTGAAAAAGCA
p5	UAS1, 2 mut cloning F	CATTGACTCCGATATCTCGC
p6	mlonc-mut 1A-T-R	CATAAGAACGACCCCAATTGCCTATGA
p7	mlonc-mut 1A-C-R	CATAAGAGCGACCCCAATTGCCTATGA
p8	mlonc-mut 1A-G-R	CATAAGACCGACCCCAATTGCCTATGA
p9	mlonc-mut 2T-A-R	ACATAAGTTCGACCCCAATTGCCTATG
p10	mlonc-mut 2T-C-R	ACATAAGGTCGACCCCAATTGCCTATG
p11	mlonc-mut 2T-G-R	ACATAAGCTCGACCCCAATTGCCTATG
p12	mlonc-mut 3C-A-R	TACATAATATCGACCCCAATTGCCTATG
p13	mlonc-mut 3C-T-R	TACATAAAATCGACCCCAATTGCCTATG
p14	mlonc-mut 3C-G-R	TACATAACATCGACCCCAATTGCCTATG
p15	mlonc-mut 4T-A R	TTACATATGATCGACCCCAATTGCCT
p16	mlonc-mut 4T-C R	TTACATAGGATCGACCCCAATTGCCT
p17	mlonc-mut 4T-G R	TTACATACGATCGACCCCAATTGCCT
p18	mlonc-mut 5T-A R	ATTACATTAGATCGACCCCAATTGCCT
p19	mlonc-mut 5T-C R	ATTACATGAGATCGACCCCAATTGCCT

p20	mlonc-mut 5T-G R	ATTACATCAGATCGACCCCAATTGCCT
p21	mlonc-mut 6A-T-R	AATTACAAAAGATCGACCCCAATTGCC
p22	mlonc-mut 6A-C-R	AATTACAGAAGATCGACCCCAATTGCC
p23	mlonc-mut 6A-G-R	CATACCCACAGCAATTACACAAGATCGACC
p24	mlonc-mut 7T-A-R	CAATTACTTAAGATCGACCCCAATTGC
p25	mlonc-mut 7T-C-R	CAATTACGTAAGATCGACCCCAATTGC
p26	mlonc-mut 7T-G-R	CAATTACCTAAGATCGACCCCAATTGC
p27	mlonc-mut 8G-A-R	CATACCCACAGCAATTATATAAGATCGACC
p28	mlonc-mut 8G-T-R	GCAATTAAATAAGATCGACCCCAATTGC
p29	mlonc-mut 8G-C-R	GCAATTAGATAAGATCGACCCCAATTGC
p30	mlonc-mut 9T-A-R	AGCAATTTCATAAGATCGACCCCAATTG
p31	mlonc-mut 9T-C-R	AGCAATTGCATAAGATCGACCCCAATTG
p32	mlonc-mut 9T-G-R	AGCAATTCCATAAGATCGACCCCAATTG
p33	mlonc-mut 10A-T-R	CAGCAATAACATAAGATCGACCCCAATTG
p34	mlonc-mut 10A-C-R	CAGCAATGACATAAGATCGACCCCAATTG
p35	mlonc-mut 10A-G-R	CAGCAATCACATAAGATCGACCCCAATTG
p36	UAS1, 2 mut cloning R	GCACCATACATAGTATAGCC
p37	mlonc-mut 1A-T-F	GGGGTCGTTCTTATGTAATTGCTGTGGGTA
p38	mlonc-mut 1A-C-F	GGGGTCGCTCTTATGTAATTGCTGTGGGTA
p39	mlonc-mut 1A-G-F	GGGGTCGGTCTTATGTAATTGCTGTGGGTA
p40	mlonc-mut 2T-A-F	GGGTCGAACTTATGTAATTGCTGTGGGTA
p41	mlonc-mut 2T-C-F	GGGTCGACCTTATGTAATTGCTGTGGGTA
p42	mlonc-mut 2T-G-F	GGGTCGAGCTTATGTAATTGCTGTGGGTA
p43	mlonc-mut 3C-A-F	GGTCGATATTATGTAATTGCTGTGGGTATG

p44	mlonc-mut 3C-T-F	GGTCGATTTTATGTAATTGCTGTGGGTATG
p45	mlonc-mut 3C-G-F	GGTCGATGTTATGTAATTGCTGTGGGTATG
p46	mlonc-mut 4T-A-F	GTCGATCATATGTAATTGCTGTGGGTATGC
p47	mlonc-mut 4T-C F	GTCGATCCTATGTAATTGCTGTGGGTATGC
p48	mlonc-mut 4T-G F	GTCGATCGTATGTAATTGCTGTGGGTATGC
p49	mlonc-mut 5T-A F	TCGATCTAATGTAATTGCTGTGGGTATGC
p50	mlonc-mut 5T-C F	TCGATCTCATGTAATTGCTGTGGGTATGC
p51	mlonc-mut 5T-G F	TCGATCTGATGTAATTGCTGTGGGTATGC
p52	mlonc-mut 6A-T-F	CGATCTTTTGTAATTGCTGTGGGTATGC
p53	mlonc-mut 6A-C-F	CGATCTTCTGTAATTGCTGTGGGTATGC
p54	mlonc-mut 6A-G-F	GGTCGATCTTGTGTAATTGCTGTGGGTATG
p55	mlonc-mut 7T-A-F	GATCTTAAGTAATTGCTGTGGGTATGCA
p56	mlonc-mut 7T-C-F	GATCTTACGTAATTGCTGTGGGTATGCA
p57	mlonc-mut 7T-G-F	GATCTTAGGTAATTGCTGTGGGTATGCA
p58	mlonc-mut 8G-A-F	GGTCGATCTTATATAATTGCTGTGGGTATG
p59	mlonc-mut 8G-T-F	ATCTTATTTAATTGCTGTGGGTATGCAATTG
p60	mlonc-mut 8G-C-F	ATCTTATCTAATTGCTGTGGGTATGCAATTG
p61	mlonc-mut 9T-A-F	TCTTATGAAATTGCTGTGGGTATGCAATTG
p62	mlonc-mut 9T-C-F	TCTTATGCAATTGCTGTGGGTATGCAATTG
p63	mlonc-mut 9T-G-F	TCTTATGGAATTGCTGTGGGTATGCAATTG
p64	mlonc-mut 10A-T-F	CTTATGTTATTGCTGTGGGTATGCAATTG
p65	mlonc-mut 10A-C-F	CTTATGTCATTGCTGTGGGTATGCAATTG
p66	mlonc-mut 10A-G-F	CTTATGTGATTGCTGTGGGTATGCAATTG
p67	SPNCRNA.1506-F	GAGTAGCCATATTGAAGAGG

p68	SPNCRNA.1506-mut-R	AGAGTCCAAGCGGATACGTGGTTTAATGAAAAC
p69	SPNCRNA.1506-R	GTTTAGGAAGCGAATTGTTGC
p70	SPNCRNA.1506-mut-F	CTTGGACTCTAAAGGGTCGATTGCGTAAAG
p71	SPBC24C6.09c-3bp_mut-R	CGTAAAGGCGGATACGTGGTTTAATGAAAA
p72	SPBC24C6.09c-3bp_mut-F	ATCCGCCTTTACGTAAAAGGGTCGATTGC
p73	fbp1 ORF F	CGCCGATACAATCAGAAGC
p74	fbp1 ORF R	CGATGAGTTTGCAGCATCC
p75	cam1 F	CTACCCGTAACCTTACAG
p76	cam1 R	TGGAAGAAATGACACGAG
p77	SPBC24c6.09c probe F	CATCGTTTTCAACGTGCATG
P78	SPBC24c6.09c probe R	ATCTTCAAGGAAAAGAGCCG
P79	SPNCRNA.1506 probe F	CTCTACTTCCAACAATGCAAC
P80	SPNCRNA.1506 probe R	GACGTTTTCATTTCGCTATCC
p81	SPBC24C6.09c mlonbox-F	CTAGTTTCAACACTTCACAAACA
p82	SPBC24C6.09c mlonbox-R	CTCTCTTTCTTTGACTCATAAA
p83	prp3 F	GCACAGTCGTTGTACAAATTCGTATTCCC
p84	prp3 R	ACGATTCTAAACGCCTCTTGTTACGATCC
p85	ade6-DSB probe2-F	CCATTTGTGGGAACCAATATG
p86	ade6-DSB probe2-R	GCTCGTTTAAATGCTAAACC
p87	SPBC24C6.09c-DSB-F	GGTTTCCCCCATCCTAATATG
P88	SPBC24C6.09c-DSB-R	GATCCACGCTCTCGAATG
P89	ade6-GSP	CATAAAGATGCAAAGTTGCACCGGG
p90	M13-F	GTAAAACGACGGCCAGT
