# Supplementary material for: lncRNA transcription induces meiotic recombination through chromatin remodelling in fission yeast
Source: Commun Biol. 2021 Mar 5;4:295. doi: 10.1038/s42003-021-01798-8 (PMC7935937; doi:10.1038/s42003-021-01798-8)
Supplement: Supplementary file 4 — Description of Additional Supplementary Files [file 42003_2021_1798_MOESM4_ESM.pdf]

Description of Additional Supplementary Files

File name: Supplementary Data 1

Description: Source data behind all graphs.
